# 

**Published:** 1847-07

**Authors:** 


### CONTENTS OF N° XLVII

OF THE

### British and Foreign Medical Review.

JULY, 1847.

34547

### PART FIRST.—Analytical and Critical Reviews.

PAGE.

ART. I.—1. Traitement Moral, Hygiène et Education des Idiots et des autres Enfants arriérés ou retardés dans leur développement, agités de Mouvements involontaires, débiles, muets non-sourds, vègues, &c. Par EDOUARD SEGUIN . 1

The Moral Treatment, Hygiene, and Education of Idiots, and of other Children backward or retarded in their development, agitated with involuntary Movements, Weak, Dumb, but not Deaf, Stammering, &c. By EDWARD SEGUIN.

2. Remarks, Theoretical and Practical, on the Education of Idiots and Children of Weak Intellect. By W. R. SCOTT, PH.D., Principal of the West of England Institution for the Education of the Deaf and Dumb . . ib.

The Education of Idiots . . . . . 4
Education of the Nervous System and of the Senses . . . 6
Moral Treatment . . . . . . . 14

ART. II.—Beiträge zur Ohrenheilkunde von Dr. WILHELM KRAMER, Sanitäts-Rath. Nebst 19 statistischen Tabellen . . . . . . 22

Contributions to Ear-Medicine. By. Dr. WILLIAM KRAMER, &c. With 19 Statistic Tables.

ART. III.—Klinische Ergebnisse gesammelt in dem Königlichen Policlinischen Institut der Universität, von dessen Assistenarzte Dr. EDUARD HENOCH, und herausgegeben von Dr. M. H. ROMBERG, &c. &c. . . . 41

Results of Clinical Observation in the Royal Polyclinical Institute of the University of Berlin. Recorded by Dr. E. HENOCH, and edited by Dr. ROMBERG.

ART. IV.—Researches into the Physical History of Mankind. By JAMES COWLES PRICHARD, M.D., F.R.S., M.R.I.A., Corresponding Member of the National Institute of France, &c. &c. Third edition. With numerous Plates . 49

2. The Natural History of Man; comprising Inquiries into the Modifying Influence of Physical and Moral Agencies on the different Tribes of the Human Family. By JAMES COWLES PRICHARD, M.D., F.R.S., M.R.I.A., Corresponding Member of the National Institute of France, &c. &c. With forty Plates and ninety Wood-Engravings . . . . . ib.

3. Appendix to the First Edition of the Natural History of Man. With six coloured Plates . . . . . . . ib.

§

PAGE

ART. V.—Recherches de Pathologie Comparée. Par CH. F. HEUSINGER . 81

Researches in Comparative Pathology. By C. F. HEUSINGER.

ART. VI.—Denkwürdigkeiten zur medicinischen Statistik und Staatsarzneikunde für Criminalisten und Aertze. Von Dr. J. L. CASPER, Professor an der Friedrich-Wilhelm's Universität, &c. &c. . . . . 91

Essays on Medical Statistics and Medical Jurisprudence, adapted for the use of Jurists and Medical Practitioners. By Dr. J. L. CASPER, Professor in the University of Berlin, &c. &c.

Influence of the Weather on Health . . . . 92
Diurnal Periods of Births and Deaths . . . . 114

ART. VII.—Die Pathologische Gewebelehre von Dr. FRIEDRICH GÜNSBURG. Erster Band. Die Krankheitsprodukte nach ihrer Entwickelung, Zusammensetzung, und Lagerung in den Geweben des menschlichen Körpers . . 118

Pathological Histology. By Dr. F. GÜNSBURG. First Volume. On the Development, Connexion, and Deposition of Morbid Products in the Tissues of the Human Body.

ART. VIII.—Neue Untersuchungen in den Gebieten der Physiologie und der practischen Heilkunde von Dr. K. H. BAUMGAERTNER . . . 128

New Researches in the Departments of Physiology and Practical Medicine. By Dr. K. H. BAUMGAERTNER.

Physiological Researches . . . . . 128
Pathological Researches . . . . . 135
Clinical Contributions. . . . . . 137

ART. IX.—Thoughts on the Nature and Treatment of several Severe Diseases of the Human Body. By E. J. SEYMOUR, M.D., F.R.S., &c. . . 139

ART. X.—The Construction and Government of Lunatic Asylums and Hospitals for the Insane. By JOHN CONOLLY, M.D., &c. . . . . 156

ART. XI.—Die Krankheiten des Zwerchfells des Menschen. Von. C. W. MEHLISS, M.D., &c. . . . . . . . . 166

The Diseases of the Diaphragm in Man. By C. W. MEHLISS, M.D., &c.

ART. XII.—1. Erste Gründe einer Physiologie der eigentlichen thierischen Natur thierischer Körper: entworfen von Dr. JOHANN AUGUST UNZER . 181

Principles of a Physiology of the Animal Nature, peculiar to Animal Bodies. By Dr. J. A. UNZER.

2. JOHANN AUGUST UNZER'S Physiologische Untersuchungen. Auf Veranlassung der Göttingischen, Frankfurter, Leipziger und Hallischer Recensionem seiner Physiologie, &c. . . . . . . . ib.

UNZER'S Physiological Inquiries: in reference to the different Reviews of his Work on the Physiology of the Animal Nature.

ART. XIII.—1. Familiar Instructions in Medicine and Surgery, with Observations on the Means of maintaining the Health of Men on Ship-board, or when employed in Unhealthy Localities. Intended for the Use of the Merchant Navy, Commanders of Yachts, Travellers, and all Persons who may be away from Medical Aid. By F. F. SANKEY, M.D. . . . . 212

2. The Scale of Medicines with which Merchant Vessels are to be furnished, in pursuance to Act 7 and 8 Vic., cap. 112; with Directions for their Use; Observations on some of the Accidents and Diseases to which Seamen are more peculiarly liable; and Directions for preserving the Health and promoting the Comfort of Merchant Seamen. By CHARLES M'ARTHUR, M.D., Surgeon Royal Navy . . . . . . . ib.

PAGE

ART. XIV.—Mental Dynamics, or Groundwork of a Professional Education. The Hunterian Oration, before the Royal College of Surgeons of England, 15th February, 1847. By JOSEPH HENRY GREEN, F.R.S. . . . 216

ART. XV.—Outlines of Structural and Physiological Botany. By ARTHUR HENFREY, F.L.S., &c., Lecturer on Botany at St. George's and the Middlesex Hospitals . . . . . . . 223

ART. XVI.—1. An Essay on the Relation of the Theory of Morals to Insanity. By T. MAYO, M.D., &c. . . . . . . 225

2. Elements of the Pathology of the Human Mind. By THOMAS MAYO, M.D., F.R.S. . . . . . . . ib.

3. Clinical Facts and Reflections: also Remarks on the impunity of Murder in some Cases of Insanity. By T. MAYO, M.D., F.R.S. . . . ib.

ART. XVII. 1. Rapport à l'Académie Royale de Médecine sur la Peste et les Quarantaines fait au nom d'une Commission. Par M. le Dr. PRUS. Accompagné de Pièces et Documents, et suivi de la Discussion dans le sein de l'Académie . . . . . . . 242

2. A Treatise on the Plague, more especially on the Police Management of that Disease, illustrated by the Plan of Operations successfully carried into Effect in the late Plague of Corfu; with Hints on Quarantine. By A. WHITE, M.D., Deputy Inspector of Military Hospitals, late Superintendent of the Plague in Corfu, &c. . . . . . . ib.

3. Correspondence respecting the Quarantine Laws, since the Correspondence last presented to Parliament. Presented by Command to the House of Commons, in pursuance of their Address of May 19, 1846. . . ib.

French Regulations . . . . . . 263

English Regulations . . . . . . ib.

ART. XVIII.—Researches on the Chemistry of Food. By JUSTUS LIEBIG, M.D., Professor of Chemistry in the University of Giessen. Edited from the Manuscript of the Author, by WILLIAM GREGORY, M.D., Professor of Chemistry in the University of Edinburgh . . . . 264

ART. XIX.—Practical Observations on some of the Diseases of the Stomach and Alimentary Canal. By JAMES ALDERSON, M.D., F.R.S., late Senior Physician to the Hull Infirmary . . . . . . 273

ART. XX.—Ueber die Gesetze nach welchen die Mischung von Flüssigkeiten und ihr Eindringen in permeable Substanzen erfolgt, mit besonderer Rücksicht auf die Vorgänge im menschlichen und thierischen Organismus. Von JULIUS VOGEL . . . . . . 276

On the Laws regarding the Mixing of Fluids and their penetration into permeable substances, with special reference to the processes in the Human and Animal Organism. By JULIUS VOGEL.

### Part Second.—Bibliographical Notices.

PAGE

Art. I.—Experimental Researches on the Post-Mortem Contractility of the Muscles, with Observations on the Reflex Theory. By Bennet Dowler, m.d. . 279

Art. II.—Practical Observations on the Pathology and Treatment of certain Diseases of the Skin, generally pronounced Incurable. Illustrated by upwards of Forty Cases. By Thomas Hunt, m.r.c.s., &c. . . 280

Art. III.—The Ancient World; or Picturesque Sketches of Creation. By D. T. Ansted, m.a., f.r.s., f.g.s.; Professor of Geology in King's College, London, &c. &c. . . . . . . 281

Art. IV.—A Lecture, introductory to a Course of Clinical Medicine, delivered in the Theatre of Queen's College, Birmingham, &c. &c. By Samuel Wright, m.d., &c. . . . . . . . 282

Art. V.—Hygrometrical Tables, to be used with, and Description of the Dry- and Wet-Bulb Thermometers. By James Glaisher, Esq., of the Royal Observatory, Greenwich . . . . . . 283

Art. VI.—Education and Educational Institutions considered, with reference to the Industrial Professions and the present aspect of Society. By the Rev. J. Booth, ll.d., f.r.s., m.r.i.a., &c., formerly Principal of Bristol College, and Vice-Principal of the Liverpool Collegiate Institution . . . 284

Art. VII.—Observations on the Connexion between Famine and Fever in Ireland and elsewhere. By Henry Kennedy, a.b., m.b., &c.. . . 285

Art. VIII.—An Account of the late Epidemic of Scarlatina in Newcastle and its Neighbourhood. By Edward Charlton, m.d., Edin. &c. . . 286

Art. IX.—Hülfstabellen zur Erkennung zoochemischer Substanzen. Von Dr. Freiherrn e. von Bibra. . . , . 486

Tabular Aids to the Recognition of Zoochemical Substances. By Dr. F. E. von Bibra.

Art. X.—Body and Soul; or Life, Mind, and Matter, considered as to their peculiar nature, and combined condition in living things; with a view to render the Physiology of Life and Mind more easily understood by the general reader. By George Redford, m.r.c.s. &c. . . . . . 287

Art. XI.—Metastasi Riprovate dalla Struttura dei Tessuti e dalle funzioni dei Medesimi. Tratto di G. B. Bellini, Maestro Operatore in Santa Maria Nuova . . . . . . . . 288

Metastasis Disproved by the Structure and Functions of Tissues. By G. B. Bellini, Operating Surgeon in the large Hospital of Florence.

